# Delila-PY, a Pipeline for Utilizing the Delila Suite of Software to Identify Potential DNA Binding Motifs

**DOI:** 10.1128/MRA.00160-21

**Published:** 2021-04-15

**Authors:** Kevin S. Myers, Michael Place, Jacek Kominek, Daniel R. Noguera, Timothy J. Donohue

**Affiliations:** a Wisconsin Energy Institute and Great Lakes Bioenergy Research Center, University of Wisconsin—Madison, Madison, Wisconsin, USA; b Department of Civil & Environmental Engineering, University of Wisconsin—Madison, Madison, Wisconsin, USA; c Department of Bacteriology, University of Wisconsin—Madison, Madison, Wisconsin, USA; Indiana University, Bloomington

## Abstract

Predicting potential DNA binding motifs is a critical part of understanding gene expression across all domains of life. Here, we report the development of Delila-PY, an easy-to-use pipeline for utilizing the Delila suite of software to identify DNA binding motifs.

## Abstract (precis)

Predicting potential DNA binding motifs is a critical part of understanding gene expression across all domains of life. Here, we report the development of Delila-PY, an easy-to-use pipeline to utilize the Delila suite to identify DNA binding motifs.

## ANNOUNCEMENT

**FIG 1:**
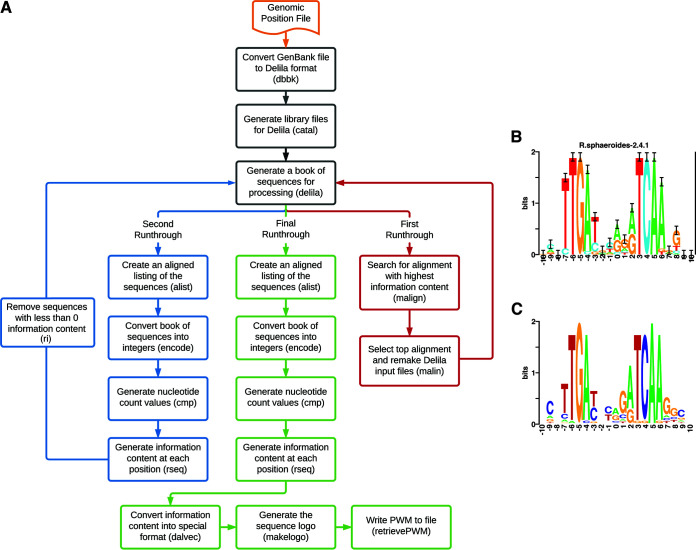
(A) Flow chart of the Delila-PY pipeline. The Delila software used at each step is indicated in parentheses. (B) Sequence logo for FnrL in R. sphaeroides from the Delila-PY pipeline. The relative heights of the letters at each position indicate the frequencies, while the overall height of the stack indicates the degree of sequence conversion, all measured in bits of information (*y* axis). The *x* axis is the relative position based on the DNA sequence coordinate used. Error bars indicate a 95% confidence interval. (C) Logo generated by WebLogo from previously identified FnrL binding sites in R. sphaeroides (12, 13). The logo and axis descriptions are the same as those for panel B.

Proteins binding to DNA to regulate transcription are a key part of growth and responding to environmental stimuli. Identification of the DNA sequences bound by these proteins can elucidate members and function of the regulons. There exist several tools that can identify these DNA motifs, including MEME (using expectation maximization) and BioProspector (using Gibbs sampling) (1, 2). The Delila suite of tools identifies motifs by maximizing information content and provides extensive flexibility in defining the parameters of motif prediction (3–7). However, the published instance of Delila requires extensive computational knowledge to install and use. Here, we present Delila-PY (Delila-PYthon), a pipeline for running the Delila programs (Fig. 1). Written in Python3 and publicly available as a Docker image (8) (recommended use case) and on GitHub, Delila-PY requires only a set of DNA sequence coordinates and a genome sequence.

Delila-PY requires the GenBank file (9, 10) for the target organism and a user-generated genomic position file with the following columns: chromosome, sequence name, strand, and genomic position (see included example files in the GitHub and Docker repositories). The genomic position file can be constructed with scripts or a spreadsheet program (e.g., Microsoft Excel) and is used to indicate the region of the genome to search for the motif. Delila-PY works with individual organisms with multiple chromosomes and mobile genetic elements and microbiomes and has been tested with both bacteria and eukaryotes. Users indicate the left and right boundaries relative to a genomic position within the genomic position file (e.g., location of the center of a chromatin immunoprecipitation sequencing [ChIP-seq] peak or transcription start site) to search for a DNA binding motif. Users can also indicate a title for the motif (the default is the species name) to keep track of multiple runs of Delila-PY.

Delila-PY processes data through the Delila software (Fig. 1A) (3–7). The pipeline starts by running the dbbk and catal programs to generate the required libraries from the GenBank file. The library and genomic position files are used by *delila* (note that *delila* is a specific program within the Delila-PY software suite) to generate a book of sequences for subsequent use. To maximize the information content in the DNA motif, the pipeline runs three cycles. The first cycle uses the malign and malin programs to search for the alignment with the highest information content among all input sequences. The results of the malin program are fed back to the *delila* program to generate an updated book file. In the second cycle, the alignment book is passed to programs (alist, encode, cmp, and rseq) to calculate the information content on each sequence. Delila-PY removes poorly matching sequences (≤0 information content, ri program). Then, a new file is generated and fed back to the *delila* program to generate an updated book file. The final cycle generates a DNA motif (alist, encode, cmp, rseq, dalvec, and makelogo). Delila-PY produces a DNA sequence logo in postscript and PDF formats, the position weight matrix (PWM) (11) values of the motif as a text file, and the individual DNA sequences used to generate the DNA motif. Each program used in the pipeline requires a specifically defined parameter file, and Delila-PY makes the default parameter files, but the user can create and use their own parameter files.

As a proof-of-concept, we used Delila-PY to identify the sequence logo for the binding site of the Rhodobacter sphaeroides transcription factor FnrL using locations from ChIP-chip data (12). The resulting sequence logo from Delila-PY (Fig. 1B) resembles the logo generated from previously identified FnrL binding sites (Fig. 1C), supporting the utility and predictive power of Delila-PY (12, 13). The files needed to run Delila-PY are available on the GitHub repository and in the Docker image. We predict that Delila-PY will allow more researchers in the life and computational sciences community to take advantage of the powerful tools within the Delila suite to identify high-quality sequence DNA motifs and sequence logos.

### Data availability.

The Delila-PY Docker image can be found on the Docker hub (recommended use; https://hub.docker.com/r/glbrc/delila). All scripts used in Delila-PY are publicly available on GitHub (https://github.com/GLBRC/delila).

## References

[1] Liu X, Brutlag DL, Liu JS. 2001. BioProspector: discovering conserved DNA motifs in upstream regulatory regions of co-expressed genes. Pac Symp Biocomput 2001:127–138. 11262934

[2] Bailey TL, Boden M, Buske FA, Frith M, Grant CE, Clementi L, Ren J, Li WW, Noble WS. 2009. MEME SUITE: tools for motif discovery and searching. Nucleic Acids Res 37:W202–W208. doi:10.1093/nar/gkp335. 19458158 PMC2703892

[3] Schneider TD, Stormo GD, Haemer JS, Gold L. 1982. A design for computer nucleic-acid-sequence storage, retrieval, and manipulation. Nucleic Acids Res 10:3013–3024. doi:10.1093/nar/10.9.3013. 7099972 PMC320671

[4] Schneider TD, Stormo GD, Yarus MA, Gold L. 1984. Delila system tools. Nucleic Acids Res 12:129–140. doi:10.1093/nar/12.1part1.129. 6694897 PMC320990

[5] Schneider TD, Stephens RM. 1990. Sequence logos: a new way to display consensus sequences. Nucleic Acids Res 18:6097–6100. doi:10.1093/nar/18.20.6097. 2172928 PMC332411

[6] Schneider TD. 2006. Twenty years of Delila and molecular information theory: the Altenberg-Austin Workshop in Theoretical Biology Biological Information, Beyond Metaphor: Causality, Explanation, and Unification Altenberg, Austria, 11–14 July 2002. Biol Theory 1:250–260. doi:10.1162/biot.2006.1.3.250. 18084638 PMC2139980

[7] Schneider TD. 2010. A brief review of molecular information theory. Nano Commun Netw 1:173–180. doi:10.1016/j.nancom.2010.09.002. 22110566 PMC3220916

[8] Merkel D. 2014. Docker: lightweight Linux containers for consistent development and deployment. Linux J. https://www.linuxjournal.com/content/docker-lightweight-linux-containers-consistent-development-and-deployment.

[9] Sayers EW, Cavanaugh M, Clark K, Ostell J, Pruitt KD, Karsch-Mizrachi I. 2019. GenBank. Nucleic Acids Res 47:D94–D99. doi:10.1093/nar/gky989. 30365038 PMC6323954

[10] Sayers EW, Beck J, Brister JR, Bolton EE, Canese K, Comeau DC, Funk K, Ketter A, Kim S, Kimchi A, Kitts PA, Kuznetsov A, Lathrop S, Lu Z, McGarvey K, Madden TL, Murphy TD, O’Leary N, Phan L, Schneider VA, Thibaud-Nissen F, Trawick BW, Pruitt KD, Ostell J. 2020. Database resources of the National Center for Biotechnology Information. Nucleic Acids Res 48:D9–D16. doi:10.1093/nar/gkz899. 31602479 PMC6943063

[11] Stormo GD. 2000. DNA binding sites: representation and discovery. Bioinformatics 16:16–23. doi:10.1093/bioinformatics/16.1.16. 10812473

[12] Dufour YS, Kiley PJ, Donohue TJ. 2010. Reconstruction of the core and extended regulons of global transcription factors. PLoS Genet 6:e1001027. doi:10.1371/journal.pgen.1001027. 20661434 PMC2908626

[13] Crooks GE, Hon G, Chandonia JM, Brenner SE. 2004. WebLogo: a sequence logo generator. Genome Res 14:1188–1190. doi:10.1101/gr.849004. 15173120 PMC419797

